# Heredity in Relation to Disease

**Published:** 1908-11-07

**Authors:** 


### Heredity in Relation to Disease.

In delivering the Harveian Oration Dr. J. A. Ormerod used a lucidity of expression and an impartiality of judgment which might be regarded as a model for scientific discussion. The impetus which has been given to the study of the phenomena of inheritance by the discovery of Mendel's paper and the work of Francis Galton and Karl Pearson caused the lecturer to devote his attention more to the "De Generatione" of Harvey than to the famous "De Motu Cordis et Sanguinis." After discussing the difficulties that beset investigation in Harvey's day, he proceeded to consider the question of the inheritance of acquired characters, including disease. The most difficult point in this question is the fact that the acceptance of Weismann's theory of the continuity of the germ plasm would seem to exclude the possibility of any such transmission. The only differences that may arise therefore in the offspring result not from any particular incidents in the life of the parents, but simply from the combination of two different germ cells. Theoretically this position might at first appear unassailable were it not known that in unicellular animals the cell is both somatic and reproductive, and that external influences must therefore affect the germ plasm. This fact has been admitted by Weismann, but he denies that any inference can be made therefrom with regard to multicellular animals. This remains, however, a moot point. Such diseases as alcoholism, diabetes, syphilis, and phthisis seem at first sight to be clear cases of inheritance. But it is denied that there can be any true inheritance of microbic disease. The infection may be either antenatal in the mother, or introduced by the sperm cells of the father, thus making the infection of a still earlier date. Yet the use of wireless telegraphy should give us pause when we require direct or material contact to produce an effect.

The application of the principles of Mendelism to disease affords a wide field for research and speculation. Many abnormal bodily conditions, such as alkaptonuria, hæmophilia, and albinism, seem to correspond to the Mendelian proportions of three to one in the second generation, where one is pure recessive, two are impure dominant, and one is pure dominant. Though it cannot yet be shown that these cases are definitely Mendelian, sufficient approximation has been reached to justify further research. The basic truth of Mendelism, the purity of the gametes, seems in certain cases to be an established fact. The possibilities of Mendelism are far-reaching. The lesson of the Andalusian fowl cannot be disregarded. If two undesirable pure breeds are crossed, a mongrel may result of entirely desirable character. Is it to be hoped, then, that the children of two tainted parents may in some proportion be perfectly healthy? The study of heredity in disease is of great interest in the department of the nervous system, and the possibility of a hitherto unrecognised force working upon the germ plasm seems not without support. If we consider the instability of the nervous system as exhibited in the "neuropathic family," we are faced with the question of its origin. Unless some modified form of Lamarckianism be adopted, we are forced to fall back on the Mendelian theory, that in the case of Friedreich's disease, for example, the disease has been in the family all the time, but as recessive, and has only appeared when certain other qualities were absent. On the whole it must be admitted that our knowledge is far too indefinite for us to formulate any fixed principle. In the case even of Brown-Séquard's epileptic guinea-pigs it is not regarded as certain that the disease was inherited, but rather that the lesion produced generally vitiated health, resulting in a form of epilepsy in the offspring. Yet, viewing the advances we have made even in the last twenty years, the outlook is hopeful. Judgment must be suspended, indeed, until more facts are collected, but this does not hinder us from believing that one day the laws of heredity will be discovered. It will probably be found that they are as simple in character as the laws enunciated by Newton.

